# Prognostic significance of Oct4 and Sox2 expression in hypopharyngeal squamous cell carcinoma

**DOI:** 10.1186/1479-5876-8-94

**Published:** 2010-10-12

**Authors:** Nan Ge, Huan-Xin Lin, Xiang-Sheng Xiao, Ling Guo, Hui-Min Xu, Xin Wang, Ting Jin, Xiu-Yu Cai, Yi Liang, Wei-Han Hu, Tiebang Kang

**Affiliations:** 1 State Key Laboratory of Oncology in South China, Cancer Center of Sun Yat-Sen University, Guangzhou 510060, China; 2 Department of Radiation Oncology, Cancer Center of Sun Yat-Sen University, Guangzhou 510060, China; 3 Department of Breast Oncology, Cancer Center of Sun Yat-Sen University, Guangzhou 510060, China; 4 Department of Nasopharyngeal Carcinoma, Cancer Center of Sun Yat-Sen University, Guangzhou 510060, China

## Abstract

**Background:**

Oct4 and Sox2 are two major transcription factors related to the stem cell self-renewal and differentiation. The aim of this study was to examine the association between Oct4 and Sox2 expression levels with both the clinicopathological characteristics and prognoses of patients with hypopharyngeal squamous cell carcinoma.

**Method:**

Tumor tissue samples from 85 patients with hypopharyngeal squamous cell carcinoma were collected, and the clinical follow-up data of these patients were recorded, and expression status of Oct4 and Sox2 were examined in these tissue samples by immunohistochemistry (IHC).

**Results:**

Oct4 expression was found to be an independent predictive factor for overall survival (*p *= 0.004) in patients with hypopharyngeal squamous cell carcinoma and was independently related to loco-regional control (*p *= 0.001). Although Sox2 expression status showed no significant association with overall survival (*p *= 0.166), disease-free survival (*p *= 0.680) or loco-regional control (*p *= 0.383), when using a subgroup analysis, the subgroup with both high Oct4 and Sox2 expression had the best prognosis (*p *= 0.000). Sox2 expression could be a potential prognostic predictor for patients with hypopharyngeal squamous cell carcinoma. Simultaneous analyses of Oct4 and Sox2 expression could be more effective in evaluating the prognoses of patients with hypopharyngeal squamous cell carcinoma.

**Conclusion:**

Oct4 expression is an independent predictive factor for patients with hypopharyngeal squamous cell carcinoma, suggesting that Oct4 expression may be a useful indicator for predicting the prognosis of hypopharyngeal squamous cell carcinoma.

## Background

Head and neck squamous cell carcinoma, including hypopharyngeal squamous cell carcinoma, is one of the most common cancers worldwide and is associated with low survival and high morbidity [1,2]. Characterized by an aggressive growth pattern and lack of obvious early symptoms, hypopharyngeal squamous cell carcinoma is a cancer with the lowest survival rates among the head and neck subsites [3,4]. Although the standard therapy of surgery plus postoperative radiation results in a 5-year survival rate of 40-50%, most patients have non-resectable tumors when they are diagnosed [5]. Interestingly, the high mortality rate of patients is mainly due to poor loco-regional control, including local tissue invasion by the primary tumor and regional lymph node involvement rather than distant metastasis [6].

The cancer stem cell (CSC) hypothesis posits that tumors may be initiated and maintained by a subset of cells that maintain or acquire stem-cell properties and that each tumor contains a small subpopulation of cells that have the ability to differentiate into multiple cell lineages and self-renew [7,8]. Indeed, cancer stem cells or cancer stem-like cells have been identified in several solid tumor types such as breast cancer and colon cancer [9,10]. This subpopulation is closely associated not only with carcinogenesis, but also with recurrence and metastasis of tumors [7]. However, there is no sufficient evidence for putative cancer stem cells in hypopharyngeal cancer, and this may be important to elucidate carcinogenesis, to analyze prognosis, and to establish new therapeutic approaches for this cancer type.

Oct4 is a major member of the POU domain transcription factors, which are required for the self-renewal characteristics and differentiation potential of pluripotent embryonic stem and germ cells [11,12]. Recent data show that cells expressing high levels of Oct4 are present in breast cancer, bladder cancer and oral squamous cell carcinoma and are associated with a worse prognosis [13-15]. Sox2 is also a major transcription factor belonging to group B of the SOX family and is essential to maintain cell proliferative potential. Unlike Oct4, Sox2 is also expressed in some mature neurons [16,17]. On one hand, Sox2 can promote the proliferation of breast cancers and gliomas [18,19]. On the other hand, elimination of Sox2 can lead to gastric cancer [20]. As a transcription factor in the Sox family, Sox2 protein must bind with other proteins, such as Oct4, to regulate DNA transcription [21,22]. In this study, we evaluated Oct4 and Sox2 expression using immunohistochemical staining of tumor tissues from patients with hypopharyngeal squamous cell carcinoma and analyzed the association between expression of Oct4/Sox2, clinicopathological characteristics and prognosis of hypopharyngeal squamous cell carcinoma.

## Methods

### Patients and tissue samples

This study was approved by the Institutional Review Board and Human Ethics Committee of Sun Yet-sen University Cancer Center. A total of 85 patients were included with histologically confirmed squamous cell carcinoma of the hypopharynx who were treated from 2002 to 2004 at the Sun Yet-sen University Cancer Center. Relevant clinical pathologic features (Table 1) were obtained from the patients' files and/or by telephone interviews with the patient or their relatives. Tumor types and histological-grade classifications were designated according to World Health Organization classification of tumors: pathology and genetics of head and neck tumors [23].

**Table 1:** The expressions of Oct4 and Sox2 and their relationships with clinicopathological characteristics.

Features	No. patients	OCT4		*P^a^*	SOX2		*P^a^*
					
		High	Low		High	Low	
Gender							
Female	1	0	1	-	1	0	-
Male	84	14	70		66	18	
Age (years)^b^							
<60	41	7	34	0.885	35	6	0.154
≥60	44	7	37		32	12	
Histological grade							
Well	34	5	29	0.572	28	6	**0.030**
Moderately	39	8	31		33	6	
Poorly	12	1	11		6	6	
SCCA^c ^(ng/ml)							
≤ 1	43	8	35	0.348	34	9	0.794
> 1	21	2	19		16	5	
TSGF^d ^(ng/ml)							
≤ 70	33	4	29	0.298	24	9	0.230
> 70	22	5	17		19	3	
T Stage							
1~2	21	3	18	0.756	16	5	0.734
3~4	64	11	53		51	13	
Cervical lymph node metastasis							
Positive	19	7	12	**0.007**	16	3	0.514
Negative	66	7	59		51	15	
TNM Stage							
I~II	7	1	6	0.871	4	3	0.143
III~IV	78	13	65		63	15	
Treatment Type^e^							
L + N	7	5	2	-	5	2	-
L + N+ R	4	1	3		3	1	
L + N + C	4	1	3		3	1	
L + N + R + C	6	0	6		2	4	
N + R	2	0	2		1	1	
N + R + C	6	2	4		4	2	
R	3	1	2		2	1	
R + C	21	2	19		20	1	
C	20	1	19		17	3	
No treatment or tracheotomy	12	1	11		10	2	

^a ^Chi-square test

^b ^Patients were divided according to the median values of age.

^c ^SCCA, Squamous Cell Carcinoma Antigen

^d ^TSGF, Tumor Supplied Group of Factor

^e ^L = Laryngectomy, N = Neck dissection, R = Radiotherapy, C = Chemotherapy

### Immunohistochemistry (IHC) staining

Immunohistochemistry was performed on 4-μm-thick routinely processed paraffin sections. Oct4 was detected using a rabbit polyclonal anti-Oct4a antibody (Cell signaling, #2890, UK, dilution 1:100). Sox-2 was detected using a rabbit polyclonal anti-Sox antibody (Cell signaling, #3579, UK, dilution 1:100). A total of 85 formalin-fixed, paraffin-embedded hypopharyngeal squamous cell carcinoma tissue samples were dried overnight at 56°C. After deparaffinization and rehydration, sections were heat-pretreated in a citrate buffer (92°C in microwave oven) and incubated in 3% H_2_O_2 _to block endogenous peroxidase activity. Then the sections were examined by immunostaining using the primary antibodies overnight at 4°C in a humidity chamber. The avidin-biotin technique was applied using DAB for visualization and hematoxylin for nuclear counterstaining. Negative controls were prepared by omitting the primary antibody. Histological and IHC evaluation were independently performed by two pathologists without knowledge of the clinicopathological outcomes of the patients. Slides with indeterminate evaluation were re-evaluated, and a consensus was reached. Briefly, each slide was examined in its entirety under a light microscope, and an initial score was assigned which represented the estimated proportion of positive tumor cells (0: none; 1: < 1/4; 2: 1/4 to 1/2; 3: 1/2 to 3/4; and 4: > 3/4). Next, an intensity score was assigned which represented the average intensity of staining of the positive tumor cells (0, none; 1, weak; 2, intermediate; and 3, strong). The proportion and intensity scores were then added to obtain a total score, which ranged from 0 to 7. Specimens were categorized into one of two groups according to their overall scores: (1) low expression, < 4 points; (2) high expression, 4-7 points.

### Statistical methods

Statistical analysis was performed using the SPSS 17.0 software package for Windows. The χ^2 ^test was used to evaluate categorical variables. Associations between clinicopathological features and immunohistochemical Oct4 or Sox2 expression were analyzed using the logistic regression model with the presence of overall survival as the dependent variable. Multivariate survival analyses were performed with the Cox regression model. Overall survival (OS) was measured from the onset of treatment to the date of death or the survival status at the last date of follow-up. The loco-regional control (LRC) was the interval from the onset of treatment to the date of recurrence. Recurrence was defined as local tissue invasion by the primary tumor or regional lymph node involvement. Disease-free survival (DFS) was defined as the interval between the onset of treatment and the date when recurrence or metastasis was diagnosed. OS, LRC and DFS probabilities were estimated by the Kaplan-Meier method and the significance of differences were assessed by the log-rank test. A P-value < 0.05 was considered statistically significant, and a P-value < 0.01 was considered strongly statistically significance.

## Results

### Clinicopathological features

Table 1 presents a summary of sex, age, tumor stage, histological grade, and the status of SCCA (Squamous Cell Carcinoma Antigen) as well as TSGF (Tumor Supplied Group of Factor). In this study, there were 85 hypopharyngeal carcinoma patients consisting of 84 males and 1 female. The median age was 60 years (range: 37-82 years). According to the 6th Edition of the International Union Against Cancer (UICC) TNM classification system, there were 7 Stage II patients, 24 Stage III patients, and 54 Stage IV patients, as shown in Table 2. Recurrences were confirmed by histopathology or visual examination and were found to have occurred in 72 patients. The median time to recurrence was 5.5 months (range 1-50 months). Seventy cancer-related deaths were reported. The median time to death was 17 months (range 0.16-74 months). The reasons for death were local recurrence (62 patients), pulmonary metastases (3 patients), hepatic or abdominal cavity metastases (3 patients) and mediastinal metastases (2 patients).

**Table 2:** The relationships between clinicopathological variables and immunohistochemical features with the overall survival.

Variables	**No**. patients	OS (%)			***P *^a^**	χ**^2^**
		
		1 y	3 y	5 y		
Age^b ^(y)						
<60	41	65.9	19.5	17.1	0.555	0.348
≥60	44	63.6	25.0	20.5		
Histological grade						
Well	34	66.7	25.0	25.0	0.996	0.008
Moderately	39	67.6	20.6	17.6		
Poorly	12	61.5	23.1	17.9		
SCCA^c ^(ng/ml)						
≤ 1	43	69.8	18.6	16.2	0.200	1.639
> 1	21	71.4	19.0	14.3		
TSGF^d ^(ng/ml)						
≤ 70	33	69.7	15.2	12.1	0.244	1.356
> 70	22	68.2	22.7	18.2		
T Stage						
1~2	21	90.5	23.8	14.3	0.303	1.061
3~4	64	56.3	25.0	20.3		
Cervical lymph node metastasis						
positive	66	63.6	18.2	15.2	0.103	2.662
negative	19	68.4	36.8	31.6		
TNM Stage						
I~II	7	100	28.5	28.5	0.678	0.173
III~IV	78	61.5	21.8	17.9		
Oct4^e^						
High Expression	14	85.7	71.4	57.1	**0.000**	15.661
Low Expression	71	60.6	12.7	11.3		
Sox2^e^						
High Expression	67	59.7	23.9	19.4	0.683	0.166
Low Expression	18	83.3	16.7	16.7		
Oct4 & Sox2					**0.000**	17.991
Both high	13	88.2	76.9	61.5		
Either high	55	54.5	10.9	9.1		
Both low	17	82.4	17.6	17.6		

^a ^Log-rank test

^b ^Patients were divided according to the median values of age.

^c ^SCCA, Squamous Cell Carcinoma Antigen

^d ^TSGF, Tumor Supplied Group of Factor

^e ^Two-sided log rank test with an overall sample size of 85 subjects (of which 71 are in group

1 and 14 are in group 2) achieves 84% power at a 0.0500 significance level to detect a difference of 0.5870 between 0.1270 and 0.7140--the proportions surviving in groups 1 and 2,respectively. This corresponds to a hazard ratio of 0.1632. These results are based on the assumption that the hazard rates are proportional.

### Follow-up outcome

The last follow-up date is Sep. 29^th^, 2009, with a median follow-up time 52 months (range 7-69.5 months). The 1-, 3- and 5-year overall survival rates (OS) were 64.7%, 22.4%, 18.8%, respectively; disease-free survival (DFS) was 24.7%, 15.3%, 12.9%, respectively. The local-regional control rates were 24.7%, 16.5%, 15.3%, respectively.

### Immunohistochemical expression of Oct4 or Sox2

**Figure 1:**
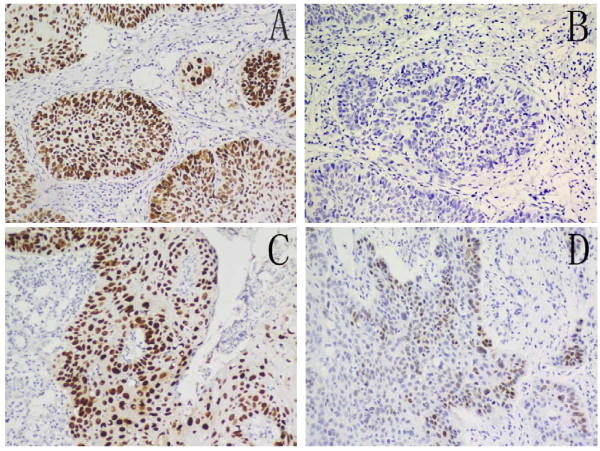
**Expression of Oct4 and Sox2**. Immunohistochemical staining for Oct4 and Sox2 expression in hypopharyngeal squamous cell carcinoma. Brown grains represent a positive signal (3, 3-diaminobenzidine staining). The positive expression site of Oct4 and Sox2 was mainly localized in the nucleus of tumor cells. (A) High Oct4 expression in tumor cells, (B) low Oct4 expression in tumor cells, (C) high Sox2 expression in tumor cells, and (D) low Sox2 expression in tumor cells.

Positive staining for Oct4 and Sox2, mainly localized in the nucleus, were observed in the cancer cells of tumor tissues (Fig. 1). The distribution of immunostaining scores is listed in the Table 1. The highest expression rate of Oct4 was 9.4% (8 of 85), whereas that of Sox2 was 71.8% (61 of 85). Furthermore, the expression Oct4 is correlated with the cervical lymph node metastasis (*p *= 0.007) whereas the expression of Sox2 is correlated with the histological grade (*p *= 0.03), as in Table 1.

### Association with prognosis

**Figure 2:**
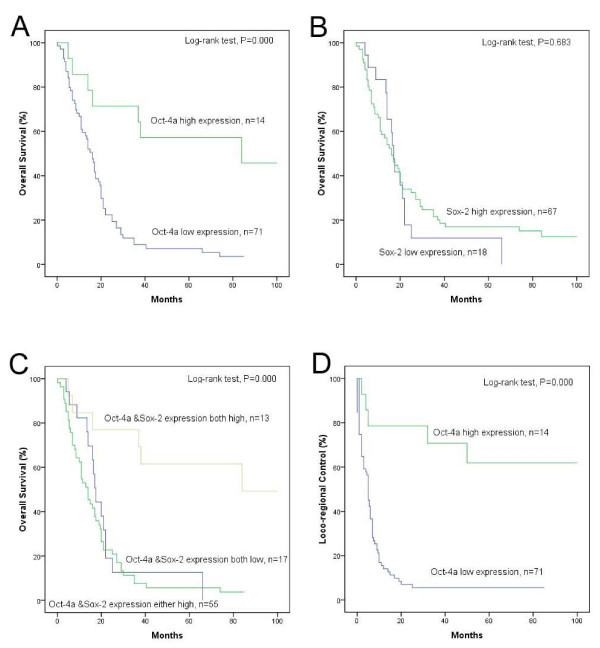
**The Kaplan-Meier survival curves**. Kaplan-Meier curves for overall survival rates according to (A) Oct4 expression status, (B) Sox2 expression status, (C) combined expression status of Oct4/Sox2 and loco-regional control rates according to (D) Oct4 expression status in hypopharyngeal squamous cell carcinoma. Statistical differences were calculated through log-rank comparisons.

Univariate analyses showed no significant association between OS, DFS or LRC and T stage, cervical lymph node metastasis, TNM stage, age, or histological grade (Table 2, 3). Patients with high Oct4 expression had a significantly better prognosis, including longer survival (*p *= 0.000) and lower recurrence rate (*p *= 0.000). Even though Sox2 expression showed no association with prognosis, the highest overall survival rate was documented in the high Oct4 expression/high Sox2 expression subgroup. The 5-years overall survival rate was 61.5% for this group (*p *= 0.000) (Fig. 2).

**Table 3:** The relationships between clinicopathological variables and immunohistochemical features with the disease-free survival and the loco-regional control.

**Variables**	**DFS**		**LRC**	
	
	***P *^a^**	**χ^2^**	***P *^a^**	**χ^2^**
			
Age^b^	0.340	0.911	0.398	0.713
Histological grade	0.852	0.320	0.782	0.492
SCCA^c^	0.265	1.243	0.165	1.932
TSGF^d^	0.352	0.868	0.370	0.804
T Stage	0.437	0.605	0.567	0.328
Cervical lymph node metastasis	0.050	3.832	0.106	2.610
TNM Stage	0.877	0.024	0.934	0.007
Oct4 expression	**0.000**	22.275	**0.000**	20.405
Sox2 expression	0.680	0.170	0.383	0.761
Oct4 & Sox2 expression	**0.000**	26.331	**0.000**	22.101

^a ^Log-rank test

^b ^Patients were divided according to the median values of age.

^c ^SCCA, Squamous Cell Carcinoma Antigen

^d ^TSGF, Tumor Supplied Group of Factor

### Multivariate analysis

A multivariate survival analysis was performed with the Cox regression model for each predictor of prognosis to calculate odds ratios, as well as 95% confidence intervals. The model was simplified in a stepwise fashion by removing variables that had a *p *value ≥0.05. Only three variables remained statistically significant as independent predictors of OS and LRC in the multivariate analysis. Also, because the variable Oct4 & Sox2 expression consisted of Oct4 expression and Sox2 expression, this variable is replaced by Sox2 expression (Table 4). The results indicate that the expression status of Oct4 (*p *= 0.004) was an independent predictive factors for prognoses of hypopharyngeal squamous cell carcinoma patients.

**Table 4:** Multivariate analysis

**Variables**	**OS**			**LRC**		
	
	**Odds ratio**	**95%CI^a^**	***P *^b^**	**Odds ratio**	**95%CI^a^**	***P *^b^**
	
Oct4 expression	0.296	0.129-0.679	**0.004**	0.183	0.040-0.475	**0.001**
Sox2 expression	0.855	0.477-1.532	0.599	1.239	0.681-2.254	0.485

^a ^CI, Confidence interval

^b ^Cox regression model

## Discussion

The relationship between cancer cells and normal stem cells is a hot topic in cell biology. There is evidence showing that some cancer cells are functionally heterogeneous which confers not only the capacity of self-renewal but also of differentiation and maturation [7,8]. This subpopulation of cancer cells may be similar to stem cells or stem-like cells. Oct4 and Sox2 have been proven to be two major transcription factors that can render an adult cell capable of being reprogrammed to become a pluripotent stem cell [12,24,25]. In addition, the expression of Oct4 or Sox2 has been reported in the cancer stem-like cells and is related to a cancer patient's prognosis. Taken together, Oct4 or Sox2 might play an important role in carcinogenesis and tumor progression and may be used as an indicator of the patient prognosis [13-15,18,19].

In the present study, we found an association between the expression of Oct4 and lymphoid metastasis, whereas the expression of Sox2 was significantly related to the histological grade of individual hypopharyngeal squamous cell carcinomas. But expression of Oct4 and Sox2 had no significant association with the T stages. More importantly, the status of Oct4 expression in tumor tissues served as a significant independent predictor of both OS and recurrence for the patients with hypopharyngeal squamous cell carcinoma. This role as an independent predictor was supported by data that patients with high Oct4 expression survived longer and had a lower recurrence rates. Although the expression of Sox2 was not associated with prognosis, the subgroup with high expressions of both Oct4 and Sox2 presented the highest 5-year overall survival rate (61.5%) of all subgroups. These data are supported by the fact that decreased expression of Sox2 might be related to the carcinogenesis human gastric epithelial cancers [26]. Thus, it may be not surprising that high expression of Oct4 could be an indicator of better prognosis for patients with hypopharyngeal squamous cell carcinoma. In fact, in mouse preimplantation embryos, Stewart CL showed that either an increase above 150% or a decrease below 50% of the endogenous Oct4 levels could serve as a trigger for the differentiation of two somatic lineages, indicating that Oct4 functions differently at lower or higher levels [27]. This may also apply for hypopharyngeal squamous cell carcinoma, as shown in this manuscript. However, the roles of Oct4 and Sox2 in hypopharyngeal squamous cell carcinoma still require further investigation.

## Conclusion

Currently, clinical TNM stage is insufficient to predict prognoses of patients with hypopharyngeal squamous cell carcinoma, patients of the same clinical stage often show different clinical course. In this study we demonstrate that Oct4 expression is an independent predictive factor for patients with hypopharyngeal squamous cell carcinoma, suggesting that Oct4 expression may be a useful indicator for predicting the prognosis of hypopharyngeal squamous cell carcinoma.

## Competing interests

The authors declare that they have no competing interests.

## Authors' contributions

WHH, NG, HXL, LG, TJ, and XYC carried out the cases collection, NG, XW and HMX carried out the immunohistochemical staining work, NG, XSX and YL analyzed results. TK and WHH conceived of the study, participated in its design and coordination and helped to draft the manuscript. All authors read and approved the final manuscript.
